# Comparison of ultrasound-guided quadratus lumborum block-2 and quadratus lumborum block-3 for postoperative pain in cesarean section: A randomized clinical trial

**DOI:** 10.1097/MD.0000000000031844

**Published:** 2022-12-09

**Authors:** Ferit Yetik, Canan Yilmaz, Derya Karasu, Nesibe Haliloğlu Dastan, Mürüvvet Dayioğlu, Çağdaş Baytar

**Affiliations:** a Gemlik State Hospital, Department of Anesthesiology and Reanimation, Bursa, Turkey; b Health Sciences University, Bursa Yüksek Ihtisas Training and Education Hospital, Department of Anesthesiology and Reanimation, Bursa, Turkey; c Gülhane Training and Research Hospital, Department of Anesthesiology and Reanimation, Ankara, Turkey; d Gazi University, Department of Anesthesiology and Reanimation, Division of Intensive Care, Ankara, Turkey; e Zonguldak Bülent Ecevit University Medicine Faculty, Department of Anesthesiology and Reanimation, Zonguldak, Turkey.

**Keywords:** analgesia, cesarean section, pain management, postoperative pain, quadratus lumborum, ultrasonography

## Abstract

The aim of this study was to compare the postoperative analgesic effects of ultrasound-guided quadratus lumborum block-2 (QLB-2) and quadratus lumborum block-3 (QLB-3) after cesarean section (C/S) under general anesthesia.

**Method:**

This was a prospective, randomized, double-blind study. A total of 143 term pregnant women with American Society of Anesthesiologists II status, who were scheduled for elective C/S under general anesthesia were randomly separated into QLB-2 and QLB-3 groups. After surgery under standardized general anesthesia, QLBs were performed with 0.3 mL.Kg^-1^ 0.25% bupivacaine in both groups. Patient-controlled analgesia (PCA) devices were used for additional analgesia. The primary outcomes were pain scores evaluated at 0, 2, 6, 12, and 24 hours. In addition, PCA demands, actual delivered tramadol doses, rescue analgesic requirements, anesthesia time, and patient satisfaction were recorded.

**Results:**

A total of 104 patients were analyzed statistically. Pain scores were statistically lower in the QLB-3 group at 2, 6, 12, and 24 hours postoperatively (*P* = .001). Anesthesia time was longer in the QLB-3 group. Patients who received QLB-3 block demonstrated significantly fewer PCA demands and lower consumption of tramadol (*P* = .003). Moreover, the first analgesic requirement time was longer along with higher patient satisfaction. In addition, all procedures were performed without any complications and side effects due to PCA were negligible.

**Conclusions:**

This study demonstrated that, although both QLBs were safe and reliable, QLB-3 provides more effective analgesia and patient satisfaction than QLB-2 in C/S.

## 1. Introduction

Cesarean section (C/S) is a surgical procedure used to deliver a baby, and majority of them are performed under regional anesthesia. General anesthesia may be preferable for an emergency C/S, where there is insufficient time to administer neuraxial anesthesia, maternal refusal or inability to cooperate with neuraxial anesthesia, contraindications to neuraxial anesthesia, failed block, and severe hemorrhage.^[1]^ Inadequate postoperative analgesia leads to delayed ambulation, predisposing to thromboembolic events, ileus, and respiratory impairment, which might lead to atelectasis and pneumonia. In addition, pain can affect breastfeeding, which may in turn may cause growth retardation in newborns. Multimodal strategies for postoperative pain control after C/S should be employed to promote rapid recovery. There are different options for pain control, such as neuraxial blocks, peripheral blocks, nonsteroidal anti-inflammatory drugs, and systemic opioids. Currently, increased ultrasound (USG) use in anesthesia practice has made it possible to safely perform central, peripheral, and plane nerve blocks. Quadratus lumborum block-2 (QLB-2) and quadratus lumborum block-3 (QLB-3) are truncal blocks used for pain relief after C/S, abdominal surgeries, inguinal hernia, and laparotomy procedures.^[2,3]^

QLB is a posterior abdominal wall block that allows the local anesthetic to spread in the paravertebral space between T4-L1. The main sonographic landmark for QLB is the quadratus lumborum muscle (QLM). QLM is surrounded by the thoracolumbar fascia (TLF) and is a bridge between the abdominal wall and lumbar paravertebral region. The QLM can be recognized by a convex probe which is transversally placed above the iliac crest in the midaxillary line. The TLF is divided into 3 layers: the anterior layer blends laterally with the transversalis fascia and medially with the fascia of the psoas major muscle. The middle layer extends between the erector spinae and QLM, while the posterior layer encloses the erector spinae muscle. TLF serves as a conduit for local anesthetic spread into the thoracic paravertebral region. Moreover, it is believed that the effects of QLB are mainly due to the thoracolumbar fascia being rich in a sympathetic network and mechanoreceptors.^[4]^ In QLB-2, a local anesthetic is injected between the posterior surface of the QLM and TLF, which separates the latissimus dorsi and paraspinal muscles. QLB-3 is also called a transmuscular QLB. The point of injection in QLB-3 is between the anterior border of the QLM and psoas major muscles. QLB is a truncal nerve block and 20 to 30 mL of local anesthetic is needed for a successful block.^[5]^

In this study, we aimed to compare the postoperative analgesic effects of USG guided QLB-2 and QLB-3 after C/S. We hypothesized that QLB-3 may be more effective for pain relief than QLB-2 after C/S.

## 2. Methods

### 2.1. Study design

This prospective, randomized, double-blind study was conducted at Health Science University Bursa Training and Research Hospital, and CONSORT guidelines were followed. Ethical approval was provided by the Ethics Committee at the Medical School of Uludağ University (May 9, 2017; reference number: 2017-7/8, ClinicalTrials.gov Identifier: NCT04733313). After obtaining informed consent, 143 parturient women who were scheduled for elective cesarean delivery under general anesthesia were enrolled in the study. All participants had an American Society of Anesthesiologists II status and term pregnancy (37–41 weeks). Exclusion criteria were an inability to comprehend, bleeding diathesis, allergy to the study agents, and localized infection. Additionally, patients whose blocks failed, who had problems with patient-controlled analgesia (PCA), and those who underwent a hysterectomy in the same setting were excluded from the study. All patients were instructed about PCA and pain scores.

### 2.2. Anesthesia management

Standard monitoring was applied to all patients. A 20-gauge intravenous cannula was inserted, and saline infusion was started. After surgical preparation, all patients were induced with propofol 2 to 2.5 mg.Kg^-1^ IV and rocuronium 1 mg.Kg^-1^ IV. After umbilical cord clamping, fentanyl 2 mcg.Kg^-1^ and 20 mg tenoxicam IV were added. Anesthesia was maintained with sevoflurane at 1 minimum alveolar concentration in a 50% oxygen-air mixture. Demographics and hemodynamic parameters were recorded. The total dose of fentanyl consumed during surgery was recorded. Patients were randomized into 2 groups: QLB-2 (n = 58) and QLB-3 (n = 62). The randomization list, sealed and opaque envelopes were prepared using a computer program before starting the study by a staff who was not included in the study. In this double-blind study, the patient and the physician who evaluated the pain scores were blind. Before extubation, an experienced anesthesiologist performed the blocks after the end of the surgery.

### 2.3. Quadratus lumborum block procedures

#### 2.3.1. Group QLB-2.

**Figure 1.**
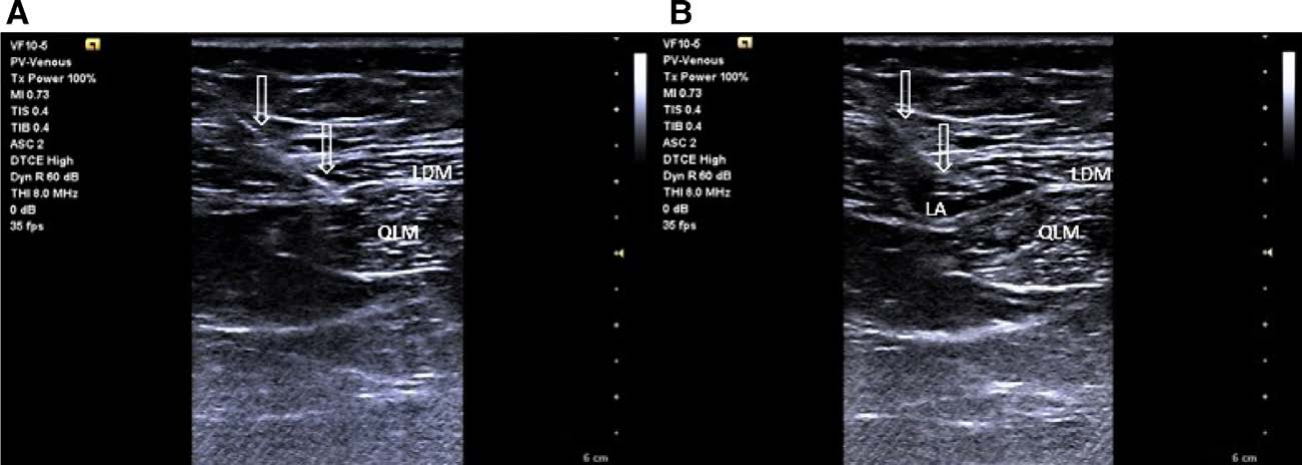
Quadratus Lumborum Block-2. (A) An ultrasound-visible block needle was inserted. (B) Local anesthetic spread middle of thoracolumbar fascia. LA = local anesthetic, LDM = latissimus dorsi muscle, QLM = quadratus lumborum muscle.

QLB 2 was performed bilaterally in the supine position using a high-frequency linear probe. Patients received 0.3 mL.Kg^-1^ of 0.25% bupivacaine (Buvasin® 0.5%, VEM, Tekirdağ, Turkey) with a block needle (80 mm, 22 G, Stimuplex®360, B. Braun, Melsungen, Germany), using an in-plane technique. The lumbar interfascial triangle, where the middle lumbar fascia joins the deep lamina of the posterior layer on the lateral border of the “erector spinae,” was targeted as the optimal point for injection (Fig. 1).

#### 2.3.2. Group QLB-3.

**Figure 2.**
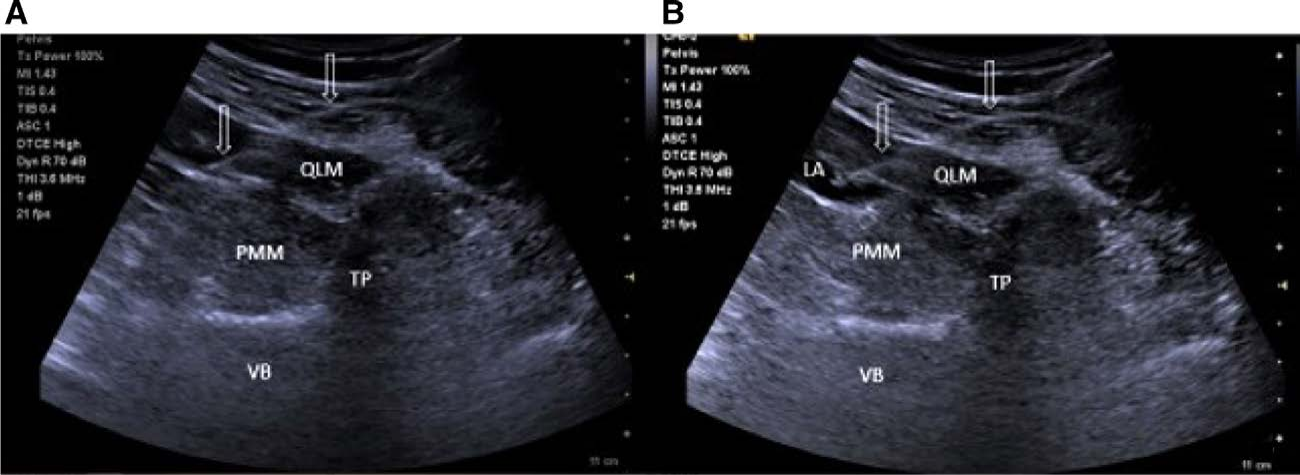
Quadratus Lumborum Block-3. (A) An ultrasound-visible block needle was inserted. (B) Local anesthetic spread anterior of thoracolumbar fascia. LA = local anesthetic, PMM = psoas major muscle, QLM = quadratus lumborum muscle, TP = transvers process, VB = vertebral body.

QLB 3 was performed bilaterally in the lateral decubitus position using a low-frequency convex probe. Patients received 0.3 mL.Kg^-1^ of 0.25% bupivacaine (Buvasin® 0.5%, VEM, Tekirdağ, Turkey) with a block needle (80 mm, 22 G, Stimuplex®360, B. Braun, Melsungen, Germany), using an in-plane technique. The targeted injection site was the anterior of the QLM at the level of its attachment to the transverse process of the L4 vertebra. After injection, the spread of the local anesthetic between the QLM and psoas major muscle was observed (Fig. 2).

### 2.4. Analgesia management

The patients were extubated after the block was performed. Intravenous PCA (CADD-Legacy® PCA, Smiths Medical, St. Paul, USA) was used for postoperative pain control. A 72 mL saline + 8 mL tramadol (50 mg/mL) IV solution was prepared. The PCA started when the patients were transferred to the recovery room. Apart from a basal infusion and loading dose, the demand dose was 25 mg Tramadol with a lockout interval of 20 min. After questioning the patients to estimate the visual analog scale (VAS) score in the recovery unit, the first bolus dose was administered to patients with a VAS score > 3 with the PCA device. The patients were discharged to the surgical ward after their vital signs were stable and the modified Aldrete score was ≥ 9. All patients received regular 1 g intravenous paracetamol at 8 hourly intervals (Partemol®, Tekirdag, Turkey). Diclofenac sodium (Diclomec®, Abdi Ibrahim, Istanbul, Turkey) 75 mg IM was administered as a rescue analgesic for patients with VAS > 5.

### 2.5. Outcomes

The primary outcome measures of the study were the visual analog scale at rest (VASR) and dynamic VAS (VASD) (0, no pain; 10 worst pain imaginable) at 0, 2, 6, 12, and 24 hours postoperatively. Secondary outcomes included the total number of PCA demands and actual doses delivered within 24 hours of the operation, anesthesia time, rescue analgesic doses, and patient satisfaction. The operation time was defined as the time from the surgical incision to the last suture. Anesthesia time was defined as the time from intubation to extubation. The satisfaction of the patients was recorded according to the postoperative pain status with a 4-point scale (very satisfied, satisfied, undecided, and not satisfied).

### 2.6. Statistical analysis

According to the results of the pilot study, the minimum sample size to be reached was found to be 38 patients for each group, with 80% power and 95% confidence interval as reference. The SPSS 24 (Statistical Package for Social Sciences, Armonk, NY) program was used for statistical analysis in the evaluation of the data obtained in the study. In the descriptive statistics of the data, mean and standard deviation were used for quantitative data, and percentage values were used for qualitative data. Kolmogorow-Smirnov normal distribution test was used in the distribution of the variables. Mann Whitney-*U* and Kruskal Wallis were used in the analysis of data that did not fit the normal distribution. Chi-square tests were used for categorical variables.

## 3. Results

**Figure 3.**
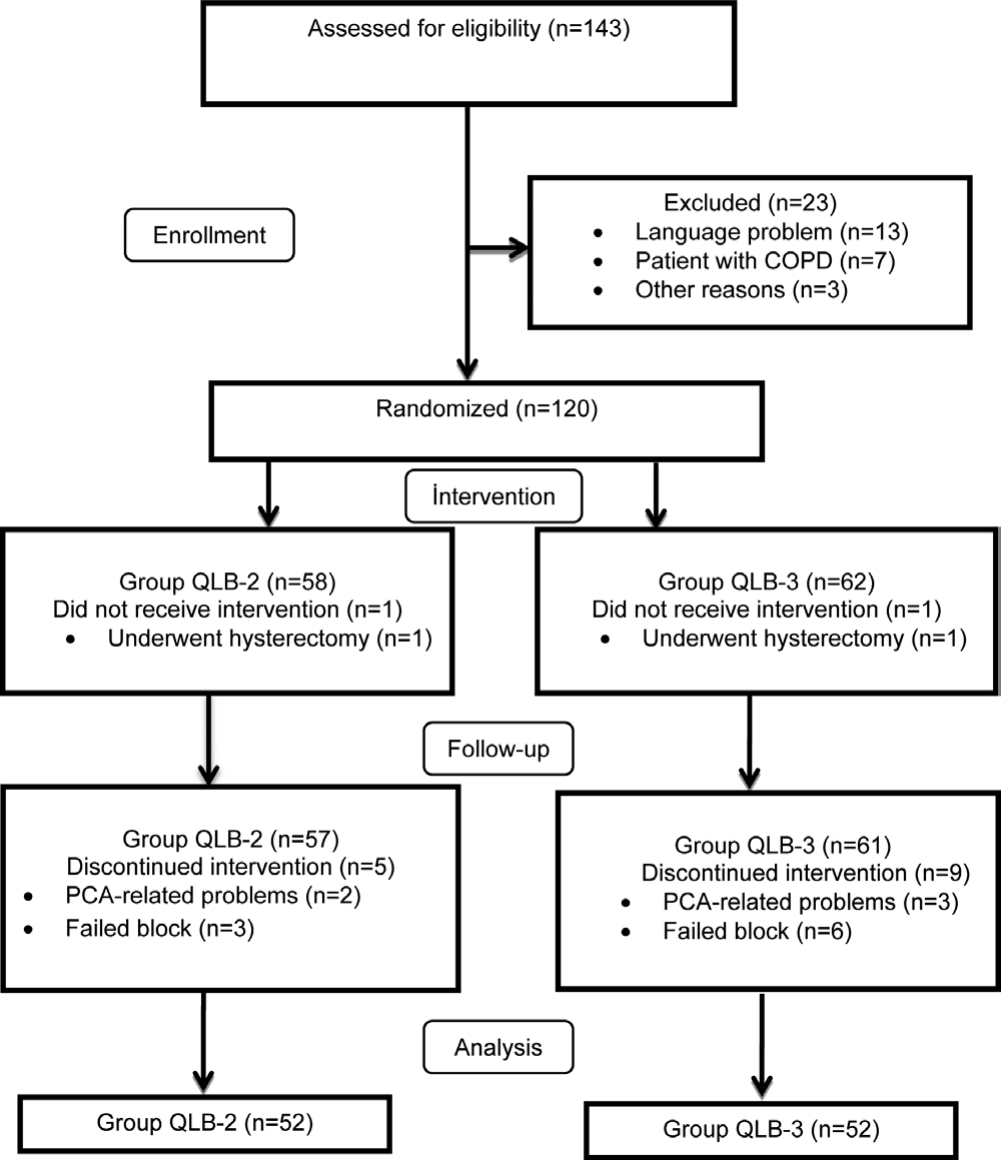
Trial flow diagram.

Of the 143 parturient women who were initially enrolled, 120 were included in the study, and 104 were statistically analyzed finally (Fig. 3).

Patients demographics were comparable with no significant differences between the 2 groups (*P* > .05, Table 1). Intraoperative fentanyl use and operation time were similar between the groups (*P* > .05). On the other hand, anesthesia time was significantly longer in the QLB-3 group (*P* < .05, Table 1).

**Table 1:** Demographic data, intraoperative fentanyl consumption, duration of surgical procedure and anesthesia.

	Grup QLB-2 (n = 52)	Grup QLB-3 (n = 52)	*P*
**Age (years**)#	29.25 ± 5.41	29.63 ± 5.83	.728
**Height (cm**)#	162.31 ± 0.48	162.10 ± 0.51	.829
**Weight (kg**)#	81.51 ± 48	80.83 ± 14.56	.819
**BMI (kg/m2**)#	30.84 ± 4.80	30.74 ± 5.20	.919
**Intraoperative Fentanyl Consumption**# **(mcg/kg**)	1.98 ± 0.04	1.98 ± 0.15	.794
**Duration of Surgical Procedure**# **(min**)	37.98 ± 6.75	37.05 ± 5.50	.434
**Duration of Anesthesia**#** (min**)	44.34 ± 6.75	47.34 ± 5.70	*.016* *

#Mean ± standard deviation.

* *P* < .05.

Patients who received QLB-3 block demonstrated significantly fewer PCA demands and lower consumption of tramadol (*P* = .003). Moreover, the first analgesic delivery from PCA was later in the QLB-3 group (*P* < .01, Table 2).

**Table 2:** Distribution of analgesic consumption by groups.

	Grup QLB-2 (n = 52)	Grup QLB-3 (n = 52)	*P*
**PCA Total Bolus** #	8.75 ± 3.071	5.19 ± 2.73	*.003* *
**PCA Total Attempts** #	9.38 ± 3.33	5.65 ± 2.97	*.027* *
**First Analgesic Time**#** (h**)	0.43 ± 0.76	1.74 ± 2.36	*.001* *
**24 h Tramadol Consumption** #** (mg**)	218.75 ± 76.83	128.46 ± 68.71	*.003* *
**Rescue analgesic**#** (mg**)	15.87 ± 34.31	8.65 ± 24.19	.218

PCA **=** patient controlled analgesia.

#Mean ± standard deviation.

* *P* < .05.

The VASR and VASD values were statistically lower in the QLB-3 group at 2, 6, 12, and 24 hours postoperatively (*P* = .001, Table 3).

**Table 3:** VASR-DVAS scores.

Time (hour)	VASR/DVAS	Grup QLB-2 (n = 52)	Grup QLB-3 (n = 52)	*P*
**0** ^**th**^	**VASR**	3.33 ± 0.78#	3.29 ± 1.03#	.831
	**DVAS**	4.08 ± 0.88 #	4.10 ± 0.99#	.917
**2** ^**th**^	**VASR**	2.62 ± 1.08#	1.40 ± 1.07#	<.001*
	**DVAS**	3.21 ± 1.14#	1.92 ± 1.23#	<.001*
**6** ^**th**^	**VASR**	1.44 ± 1.08 #	0.54 ± 0.77 #	<.001*
	**DVAS**	1.98 ± 1.07#	0.62 ± 0.84#	<.001*
**12** ^**th**^	**VASR**	1.00 ± 0.81 #	0.27 ± 0.44 #	<.001*
	**DVAS**	1.35 ± 1.06#	0.29 ± 0.49 #	<.001*
**24** ^**th**^	**VASR**	0.77 ± 0.70 #	0.19 ± 0.39 #	<.001*
	**DVAS**	1.10 ± 0.9#	0.23 ± 0.46 #	<.001*

# Mean± standard deviation.

* *P* < .05.

DVAS = dynamic visual analog scale, VASR = resting visual analog scale.

Our analyses showed no differences between the 2 groups in terms of the postoperative side effects (nausea, vomiting, insomnia, dizziness, respiratory depression, and sedation) (*P* > .05).

Although patient satisfaction after 24 hours was high in both groups, it was significantly higher in the QLB-3 group (*P* = .004).

## 4. Discussion

In this double-blind, randomized, prospective study, we studied the postoperative analgesic effect of QLB-2 and QLB-3. We found that the patients who received QLB-3 had significantly lower VASR and VASD scores, except for the 0th hour (*P* = .001). In addition to a longer time period before the first analgesic requirement, tramadol consumption and PCA demands were lower in the QLB-3 group. Additionally, patient satisfaction was significantly higher in the QLB-3 group. However, anesthesia time was significantly longer in the QLB-3 group (*P* = .016).

With increasing USG usage improving the success rates, truncal blocks have become an important component of multimodal analgesic management. Currently, a combination of opioid analgesics, non-opioid analgesics, and nerve or truncal blocks is widely used for postoperative pain relief.^[6]^ Multimodal strategies have less side effects due to lower doses as well as decreased hospitalization period and costs.^[7]^ Therefore, they are routinely recommended for postoperative analgesia.^[8]^ Truncal blocks are preferred for postoperative pain relief after C/S. Since Dr Rafael Blanco’s first description in 2007,^[9]^ QLB has become a reputable method for postoperative pain management after abdominal surgeries. The mechanism of pain relief by QLB has not yet been clarified, but has been suggested that the analgesic effect of QLB is due to the spread of the local anesthetic along the TLF and endothoracic fascia into the paravertebral space. Other theories are based on the mechanoreceptors and sympathetic nervous network along the TLF.

It is known that both QLB-2 and QLB-3 are effective analgesic procedures. Kadam^[3]^ showed first that QLB results in low pain scores and decreased opioid use. Blanco et al^[10]^ demonstrated the effectiveness of QLB-2 after C/S in a double-blind, randomized, controlled clinical study. In another study, the same authors found that QLB -2 was more effective in reducing PCA demands and the total morphine dose than transversus abdominis block (TAPB) after C/S.^[11]^ In a pediatric case series of 5 pyeloplasties, QLB-3 was shown to be an effective analgesic method.^[12]^ Similarly, Öksüz et al^[13]^ compared the analgesic effects of TAPB and QLB-3 in pediatric patients who underwent lower abdominal surgery, and found QLB-3 to be more effective. In a cadaveric case series, Elsharkawy et al^[14]^ demonstrated that the dye spreads more widely and rapidly into the paravertebral area in QLB-3 compared to QLB-2. We found that the patients who received QLB-3 had significantly lower VASR and VASD scores, except for the 0th hour. Besides a longer time period for the first analgesic requirement, the PCA demand and total tramadol consumption were both lower in the QLB-3 group. These findings support the paravertebral space theory as the main reason for the effectiveness of the blocks.^[4]^ It is also speculated that the local anesthetics administered in QLB-3 may spread through the transversalis fascia and iliac fascia to affect the nervus femoralis and lumbar plexus.

Ishio et al^[15]^ compared QLB-2 and control groups, and found no significant difference in terms of duration of anesthesia and operation between them. In another study that compared TAPB and QLB-2, there were no differences between the groups in terms of the operation time, although the anesthesia time was not evaluated.^[16]^ In our study, although the operation times were similar between the 2 groups, anesthesia time was found to be longer in QLB-3. This may be explained by the difficulty in performing QLB-3 because of using curve probe, technical difficulty of imaging the necessary anatomical structures and the time required to turn patients in the lateral decubitus position. In addition, while in the QLB-3 group the failed block rate was 6/61 (9.8%), in the QLB-2 group was 3/57 (5.2%) in our study. These ratios also support this situation.

USG guided QLB is a safe procedure in terms of puncture of the peritoneum, visceral organs, and large vessels, which is associated with blind methods. Rarely, there may be unwanted femoral nerve block due to spread of the local anesthetic in the lumbar paravertebral or transversal and iliac fascia, causing weakness of the quadriceps.^[17,18]^ In our study, no complications were observed in both the groups.

In this study, we used tramadol PCA as a component of multimodal analgesia. Sedation, respiratory depression, and desaturation were not observed in either group. Despite higher tramadol doses, the nausea and vomiting scores were similar in both groups. The reason for this is that the total tramadol doses were lower than the daily suggested intravenous doses. In their study, Blanco et al^[11]^ found similar nausea and vomiting scores in both the TAPB and QLB groups. Ishio et al^[15]^ demonstrated higher nausea and vomiting scores in the general anesthesia group compared to the QLB group. Therefore, these studies support our results. Patient satisfaction, another parameter we evaluated, was higher in the QLB-3 group compared to the QLB-2 group after 24 hours. We consider that this was due to the low pain scores.

The limitations of our study include not evaluating the dermatome levels after the administration of the blocks, as well as limiting the follow-up of the postoperative analgesic effect to only the first 24 hours. Therefore, the long-term results could not be compared. Another limitation is that the blocks could not be performed preoperatively because of the concern that the fetus might be affected by the local anesthetic drugs.

## 5. Conclusion

Our study showed that QLB-3 is more effective than QLB-2 after C/S. Anesthesia time was statistically longer but could be ignored when we consider the lower pain scores and reduced tramadol consumption. Our study also suggested that both QLBs were safe and reliable blocks for C/S.

## Author contributions

**Conceptualization:** Ferit Yetik, Canan Yilmaz.

**Data curation:** Ferit Yetik, Nesibe Haliloğlu Dastan, Çağdaş Baytar.

**Formal analysis:** Derya Karasu, Çağdaş Baytar.

**Investigation:** Canan Yilmaz, Derya Karasu.

**Methodology:** Ferit Yetik.

**Project administration:** Canan Yilmaz.

**Software:** Canan Yilmaz, Mürüvvet Dayioğlu.

**Validation:** Mürüvvet Dayioğlu.

**Visualization:** Derya Karasu, Mürüvvet Dayioğlu.

**Writing – original draft:** Ferit Yetik, Çağdaş Baytar.

**Writing – review & editing:** Ferit Yetik, Canan Yilmaz.
